# The Defect Passivation of Tin Halide Perovskites Using a Cesium Iodide Modification

**DOI:** 10.3390/molecules28176414

**Published:** 2023-09-03

**Authors:** Linfeng He, Jin Cheng, Longjiang Zhao, Xinyao Chen, Xiaoping Zou, Chunqian Zhang, Junming Li

**Affiliations:** 1 Beijing Key Laboratory for Sensor, School of Applied Science, Beijing Information Science and Technology University, Beijing 100101, China; 2021021047@bistu.edu.cn (L.H.); chenxinyao0206@163.com (X.C.); xpzou2014@163.com (X.Z.); zhangcq@bistu.edu.cn (C.Z.);; 2 College of Engineering, Qufu Normal University, Rizhao 276826, China; honghuzlj@qfnu.edu.cn; 3 School of Instrument Science and Opto-Electronics Engineering, Beijing Information Science and Technology University, Beijing 100101, China; 4 Fujian Key Laboratory of Electrochemical Energy Storage Materials, Fuzhou University, Fuzhou 350002, China

**Keywords:** cesium iodide, fill factor, stability, lead-free, tin-based perovskite

## Abstract

Tin-based perovskites are promising for realizing lead-free perovskite solar cells; however, there remains a significant challenge to achieving high-performance tin-based perovskite solar cells. In particular, the device fill factor was much lower than that of other photovoltaic cells. Therefore, understanding how the fill factor was influenced by device physical mechanisms is meaningful. In this study, we reported a method to improve the device fill factor using a thin cesium iodide layer modification in tin-based perovskite cells. With the thin passivation layer, a high-quality perovskite film with larger crystals and lower charge carrier densities was obtained. As a result, the series resistance of devices was decreased; the shunt resistance of devices was increased; and the non-radiative recombination of devices was suppressed. Consequently, the fill factor, and the device efficiency and stability were greatly enhanced. The champion tin-based perovskite cells showed a fill factor of 63%, an efficiency of 6.1% and excellent stability. Our study reveals that, with a moderate thin layer modification strategy, the long-term stability of tin-based PSCs can be developed.

## 1. Introduction

Today, halide perovskite solar cells (PSCs) have achieved 26.1% certificated power conversion efficiency (PCE) and their stability also meets the main requirements of international standards for terrestrial photovoltaic (PV) modules [1,2]. Therefore, they are emerging as competitive candidates for the next generation of photovoltaic techniques. Typically, the halide perovskites for PV have an ABX_3_ formula where A is a monovalent cation formamidinium (CH_3_(NH_2_)_2_^+^, FA^+^), methylammonium (CH_3_NH_3_^+^, MA^+^) or cesium (Cs^+^); B is a divalent metal lead (Pb^2+^) or tin (Sn^2+^); and X is an anion chlorine (Cl^−^), bromine (Br^−^), or iodine (I^−^). So far, all high-performance PSCs have been achieved using lead-based perovskites as the active absorber layer, and the toxicity of the lead element has caused environmental pollution and health concerns [3]. Tin-based perovskites, as non-toxic perovskites, have the potential to outperform the PCE and stability of their counterpart lead perovskites, due to their excellent photophysical properties, such as an ideal bandgap for Shockley–Queisser limiting (1.34 eV), low exciton binding energy (55 meV), high carrier mobility (up to 75 cm^−2^/V·S), high light-harvesting ability (higher than 3 × 10^−4^/cm), etc. [4,5,6]. However, today tin perovskite cells typically show low performance, in particular, a much lower fill factor compared with lead perovskite cells or silicon photovoltaic cells [7]. To further improve the performance of tin-based PSCs, the fill factor needs significant improvement and more physical insights. In theory, tin-based perovskite solar cells could achieve a maximum fill factor limited to 90%, due to the inevitable loss from radiative recombination (according to the Shockley–Queisser model). Besides that, the fill factor of tin perovskite cells is mainly affected by three processes: (i) the overall Ohmic series resistance in the device; (ii) the non-radiative recombination process in the device and iii) the charge extraction ability in the device [8]. Moreover, tin-based perovskites have a relatively low defect formation energy threshold during thin film preparation. And the surface is the place where defects are most easily formed. These defects induce inefficient charge extraction of the cells, non-radiative carrier recombination or current leakage through shunts, which could result in a low device fill factor. Thus, the passivation of surface defects is inevitable to improve the performance of tin-based perovskite cells.

Recently, several strategies have been adopted to improve the fill factor in lead-based perovskite cells, such as inserting a thin passivation layer, smoothing the perovskite film, utilizing the prepatterned charge transport layer or chemical doping to strengthen the conductivity of charge transporting materials, etc. [9,10,11]. These strategies give an effective guide to improving the performance of tin-based perovskite solar cells. And in lead-based perovskite cells, the inorganic cesium cation can effectively enhance the device performance, which has been confirmed in mixed FA/Cs and FA/MA/Cs perovskite cells [12,13,14,15,16]. Xu et al. reported a CsI-SnO_2_ complex electron transport layer as a buried interface strategy: the CsI modification facilitates the following perovskite layer growth; reduces the interfacial defects; and provides a more suitable energy level alignment between the electron transport layer and perovskite layer. Therefore, the PCE was significantly increased and the resistance against UV illumination was enhanced [12]. Wang et al. applied a thin CsI layer on the top and bottom of the perovskite layer; the application of the double CsI layer greatly improved the perovskite morphology and the device exhibited enhanced device PCE and stability [13]. Rajendran et al. doped CsI in a SnO_x_ electron transport layer as a bulk and surface passivation strategy; a lower trap-filled limit voltage, lower trap density and suppressed recombination at interface were observed in the perovskite device; therefore, the efficiency was greatly improved [14]. Hu et al. doped the CsI in hole transporting material NiOx; the conductivity was increased and the surface trap densities were reduced; meanwhile, ion immigration in the perovskite film was suppressed. As a result, the device showed a greatly enhanced efficiency and remarkable fill factor [15].

Since one important strategy for tin-based PSC development is learned from its lead counterpart, guided by this, here we developed an inorganic halide salt, cesium iodide (CsI), as a passivation layer between the tin perovskites (FA_0.75_MA_0.25_SnI_3_) and electron transporting layer to enhance the device performance. The thin CsI layer reduced the interfacial defects, therefore reduced the charge transport losses, and finally increased the fill factor and the device stability. In particular, with the thin CsI layer passivation, the device performance was not decreased in the first 900 h.

## 2. Results and Discussion

**Figure 1:**
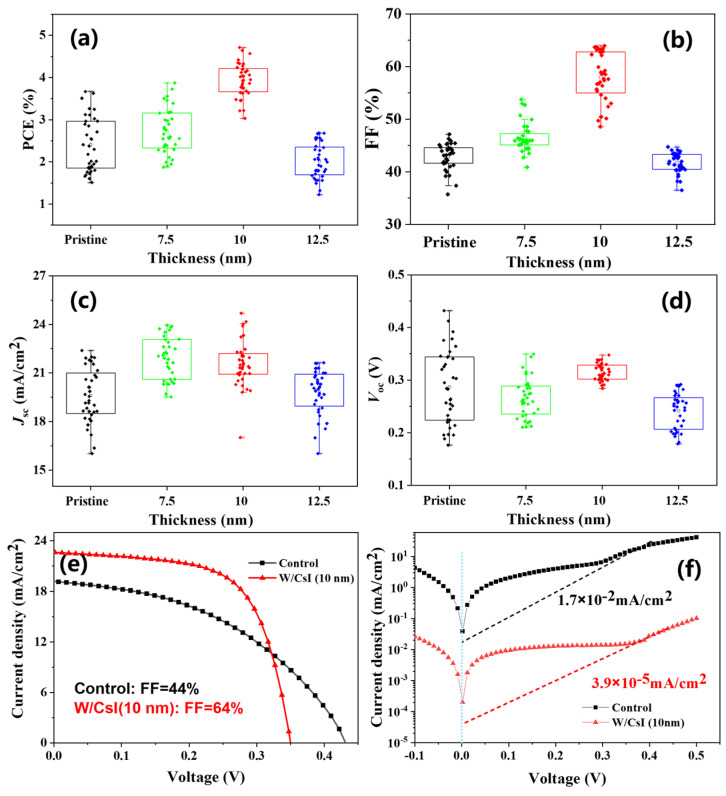
Photoelectric performance of tin-based PSCs with varied CsI thickness. (**a**–**d**) Statistical box chart: fill factor, short-circuit current density, open-circuit voltage and power conversion efficiency. (**e**,**f**) The light and dark *J*-*V* curves of champion devices without/with CsI (10 nm).

To evaluate the effect of the CsI layer on device performance, a set of inverted structure PSCs with/without CsI layer were fabricated under the configuration ITO/PEDOT:PSS/PVSK/CsI (0, 7.5, 10 and 12.5 nm)/C_60_/BCP/Ag, where PVSK represents the light absorber layer FA_0.75_MA_0.25_SnI_3_; PEDOT:PSS represents Poly(3,4-ethylenedioxythiophene)-poly(styrenesulfonate) and BCP represents for bathocuproine (as shown in Figure S1). The statistics data for the fill factor (FF), short-circuit current density (*J*_sc_), open-circuit voltage (*V*_oc_) and PCE of PSCs (18 of each batch) are presented in Figure 1a. The reference devices (without CsI layer) showed an average PCE of 2.4%, with an FF of 42.9%, *J*_sc_ of 19.6 mA/cm^2^ and *V*_oc_ of 0.29 V (in average). When the thin CsI film was inserted between the perovskite and C_60_ layers, the device performance was firstly increased. In particular, a device with a 10 nm CsI layer exhibited an average PCE of 3.9%, with an FF of 58.0%, *J*_sc_ of 21.57 mA/cm^2^ and *V*_oc_ of 0.32 V (in average). When the CsI thickness (12.5 nm) was further increased, the device performance slightly decreased. The improved PCE of PSCs can mainly be attributed to the significant improvement in the fill factor from 43% to 58% (on average). Figure 1b showed the current density–voltage (*J-V*) curves of champion PSCs with/without the thin CsI layer. The champion PSCs with 10 nm CsI showed an increased PCE of 4.71%, with an FF of 64%, *J*_sc_ of 21.81 mA/cm^2^ and *V*_oc_ of 0.34 V. In contrast, the champion control device exhibited a PCE of 3.67% (FF = 44%, *J*_sc_ = 19.15 mA/cm^2^ and *V*_oc_ = 0.43 V) (Figure S2). The resulting photovoltaic parameters of the above PSCs are summarized in Table 1.

The improved performance of 10 nm CsI modified tin-based perovskite solar cells (CsI-modified PSCs) can be partially explained by the change in series resistance (*R*_s_) and shunt resistance (*R*_sh_), as shown in Table 1. The *R*_s_ originated from the internal resistance in PSCs, more specifically, the resistance due to the light-absorbing material, interface barriers, charge collecting interlayers and metal electrodes. The *R*_sh_ was accounted for by the leakage paths in PSCs, for example, the pinholes in photoactive layer and recombination losses [17,18], as follows:

(1) $$J_{SC}=J_{ph}-J_{0}\left[exp\left(\frac{q\left(V-JR_{s}\right)}{nkT}\right)-1\right]-\frac{JR_{s}}{R_{sh}}$$

(2) $$V_{oc}=\left(\frac{nkT}{q}\right)ln\left\{\frac{J_{ph}}{J_{0}}\left(1-\frac{V_{oc}}{J_{ph}R_{sh}}\right)\right\}$$

where $J_{ph}$ denotes the photocurrent density; $J_{0}$ denotes the reverse bias saturation current density; q denotes the elementary charge; $R_{s}$ denotes the series resistance; $R_{sh}$ denotes the shunt resistance; k denotes the Boltzmann’s constant; and T denotes the temperature. For the ideal case, $R_{s}$ should be close to zero and $R_{sh}$ should be close to infinity. We found that $R_{s}$ for CsI-PSCs was 33.4 Ω, much lower than that of the control device (99.1 Ω). The reduced series resistance would benefit from the increase in the device fill factor. And the $R_{sh}$ for CsI-PSCs was 1451.9 Ω, much higher than that of the control device (955.4 Ω). Moreover, the reverse saturation current density from dark *J-V* was calculated to be 1.7 × 10^−2^ mA/cm^2^ for the control device and 3.9 × 10^−5^ mA/cm^2^ for the CsI-PSCs (Figure 1d). The significant reduction in reverse saturation current density indicates a lower carrier density, which would decrease the current leakage and reduce the charge recombination in the CsI-modified PSCs, therefore being beneficial for the device fill factor increase [19,20].

**Figure 2:**
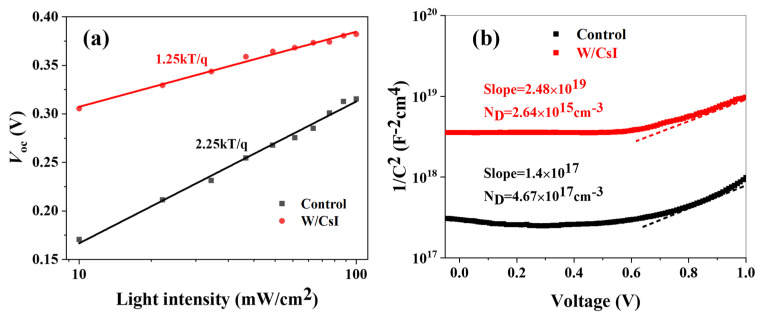
The charge carrier transfer process in tin perovskite samples: (**a**) *V*_oc_ versus light-intensity plots of tin PSCs; the light intensity ranges from 10 to 100 mW/cm^2^. (**b**) Capacitance–voltage curves of perovskite films.

The effect of the thin CsI layer on non-radiative recombination loss was studied with a light intensity-dependent experiment on solar cells, as shown in Figure 2a. The data fitting yielded a slope of 2.25 for control cells and a much lower 1.25 for CsI-modified PSCs. Slope = 1 indicates no trap-assisted recombination losses and >1 indicates that the trap-assisted recombination losses dominate [21,22]. Thus, the CsI-modified PSCs showed relatively low non-radiative recombination loss, which could be one reason for the improved fill factor. Meanwhile, considering the carrier accumulation at the perovskite/ETL interface would affect the carrier extraction and the capacitance; the capacitance–voltage curve was characterized for perovskite film by a configuration of ITO/PEDOT:PSS/PVSK/(without/with 10 nm CsI)/Ag (Figure 2b and Figure S3). The charge carrier densities of the perovskite films were derived from the slope of the capacitance–voltage (Mott–Schottky) plot following [19,23]:

(3) $$slope=\frac{2}{\varepsilon \varepsilon_{0}A^{2}eN_{D}}$$

where ε denotes the dielectric constant of the perovskite; $\varepsilon_{0}$ denotes the permittivity of the free space; A denotes the area; e denotes the elementary charge; and $N_{D}$ denotes the charge carrier density. The pristine film has a carrier density of 4.67 × 10^17^ cm^−3^. For the perovskite/CsI film, the charge carrier density was decreased by roughly two orders of magnitude to 2.64 × 10^15^ cm^−3^. The reduced background carrier density could lower the leakage current, therefore improving the fill factor in CsI-modified PSCs [24].

**Figure 3:**
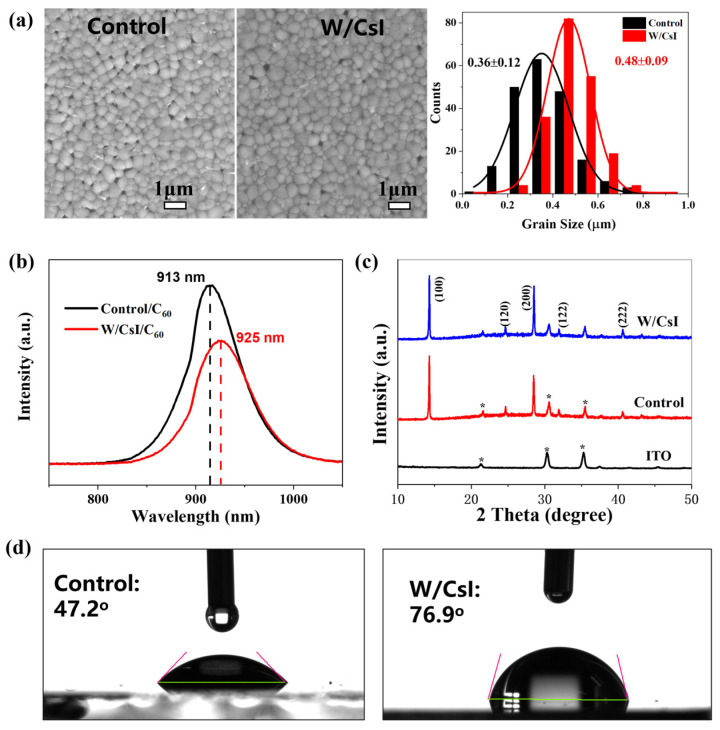
Morphology and crystallization of the perovskite films without CsI layer (control) and with CsI layer (W/CsI) modification. (**a**) Top-view SEM images of the surface morphology, left: control; middle: with 10 nm CsI; and right grain size distribution curves of control film and perovskite/CsI (10 nm) films. (**b**) Steady-state PL spectra. (**c**) XRD patterns of the control film and perovskite/CsI film (10 nm) and * represents the peak of ITO (**d**) Contact angle measurement, left: control, right: with 10 nm CsI.

The morphology of perovskite film is crucial for the device performance; therefore, to gain more insights into the improved fill factor, we examined the film morphology with scanning electron microscopy (SEM). The average size of perovskite crystals was increased from 360 nm (control film) to 480 nm (perovskite/CsI (10 nm) film) (Figure 3a and Figure S4). The inorganic small and oxidation-stable cesium cation could embed into the FA_0.75_MA_0.25_SnI_3_ perovskite surface (Figure S5) and then assist the crystal growth to its optimal phase. On the other hand, it is known that CsSnI_3_ perovskite crystals are formed at room temperature and do not need an annealing and antisolvent process; thus, it is possible that the CsI would chemically react with unpaired/unreacted SnI_2_ on the perovskite surface to form the complex Cs/FA/MA-based perovskites crystals. The radius of the Cs+ cation was smaller than that of the FA+ or MA+ molecules. According to the Goldschmidt tolerance factor $t=\frac{r_{A}\;+{\;r}_{B}}{\sqrt{2(r_{B}\;+{\;r}_{X})}}$, where $r_{A}$, $r_{B}$ and $r_{X}$ are radii of A (FA^+^,253 pm; MA^+^ 217 pm; Cs^+^,169 pm), Sn^2+^ (141 pm) and I^−^ (220 pm), respectively, the t value of FA/MA/Cs-based perovskites is closer to 1, which indicates greater structure structural stability. The existence of FA/MA/Cs-based perovskite deductions was in accordance with the photoluminance (PL) spectra (Figure 3b and Figure S5). The PL peaks for SnF_2_-containing FASnI_3_, MASnI_3_ and CsSnI_3_ were 890 nm [25], 953 nm and 955 nm [26], respectively. The PL peaks showed a small red-shift after the insertion of a thin CsI film between the perovskite and C_60_ layers. Meanwhile, after CsI passivation, the PL intensity of the perovskite/CsI/C_60_ layer was much lower than that of the perovskite/C60 layer (Figure 3b). When the perovskite layer was excited by photons, holes/electrons were formed; then, the photo-generated electrons were extracted and transferred to the electron transport layer through the perovskite/electron transport layer interface. The PL quenching means the photo-generated electrons were more efficiently extracted and transferred across the interface in the CsI modification sample. Thus, the left photo-generated electrons for the photoluminescence were reduced and therefore the light intensity was lower. Consequently, the non-radiative recombination process would be reduced in CsI-modified PSCs, which means the device fill factor and the device current would be increased.

We further analyzed the film crystal structure using X-ray diffraction (XRD) patterns (Figure 3c and Figure S5). Both perovskite and perovskite/CsI films showed almost identical results; there were two strong peaks at about 14.1° and 28.2°, corresponding to the (100) and (200) crystal planes of FASnI_3_, which confirm an orthorhombic crystal structure and the preferred growth orientation [27]. We would like to point out that, in the PVSK/CsI sample, the quantity of CsSnI_3_ should be too weak to be characterized using the XRD technique. To further explore the moisture stability of the thin films, the sample was characterized by contact angle measurement, in which 5 microliters of water was dripped onto the sample surface. The perovskite/CsI film showed a relatively hydrophobic contact angle of 76.9°, whereas the control perovskite film showed a contact angle of 47.2° (Figure 3d and Figure S7). This was caused by the hydrophobic inorganic CsI cover on the perovskite layer. The improved hydrophobic property would prevent the moisture seeping into the perovskite/CsI layer, which was beneficial to the film and device stability.

**Figure 4:**
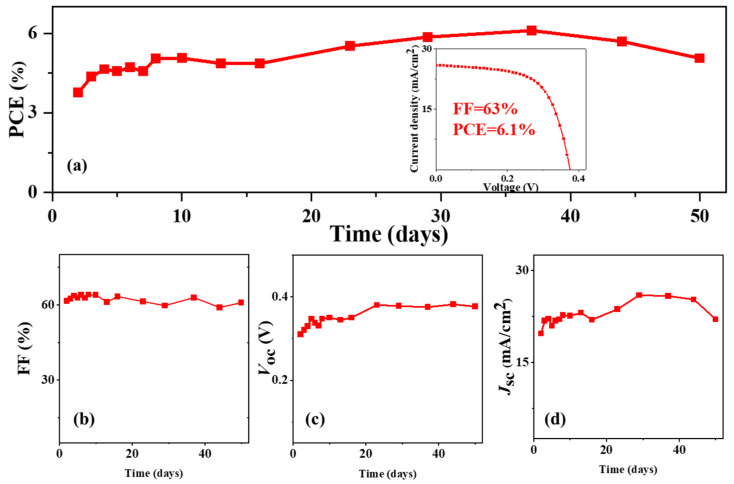
Evolution of photovoltaic parameters of the champion CsI-modified PSCs: (**a**) PCE variation; the inset shows the *J-V* curves of the CsI-modified PSCs at 40 days; (**b**) fill factor variation; (**c**) *V*_oc_ variation; (**d**) *J*_sc_ variation.

Poor device stability of tin-based perovskite cells is another challenge in their application. In our study, the unencapsulated tin-based perovskite cells were stored in a nitrogen glovebox to investigate their stability. For the control devices, the cells were degraded to a yellow color and completely lost their photovoltaic characteristic after 900 h storage. With the thin CsI layer passivation, the device performance was not decreased in first 900 h, as shown in Figure 4. In more detail, the PCE showed a continuous increase from 3.8% to 6.1% (Figure 4a); the fill factor showed a slight increase from 61.6% to 62.9% (Figure 4b); the *V*_oc_ showed an increase from 0.31 to 0.38 V (Figure 4c); and *J*_sc_ showed an increase from 19.7 to 25.9 mA/cm^2^ in the first 700 h and slightly decreased to 25.8 mA/cm^2^ at 900 h (Figure 4d). This stability result was similar to our previously reported study [28]. In our previous study, the tin-based perovskite CsSnI_3_ film was prepared using a vacuum flash-assisted solution processing (VASP) technique and the device PCE showed a gradual increase during 180 days’ storage. The same PCE increasement trend was also reported in a study published elsewhere, i.e., Jokar et al. doped a small amount of ethylenediammonium diiodide (EDAI_2_, 1%) in a FASnI_3_ precursor solution and the device PCE gradually increased during 1462 h storage [29]. Wang et al. introduced gallic acid (GA, 1%) into a FASnI_3_ precursor solution and the device efficiency showed a slowly increasing efficiency during 1500 h storage [30]. These continuously improved device performance phenomena may have resulted from the slow passivation process: the slow passivation of film defects and gradual adjustment of crystal strains to their optimal phase in the presence of the CsI modification layer [28,29,30]. Previous research studies have reported that the tin-perovskite cells were not stable due to the easy oxidation of Sn^2+^ to Sn^4+^, the low formation energy of tin vacancies and a high level of p-doping [31]. Our study reveals that, through moderate interface engineering, stable tin-based perovskite solar cells can be achieved.

## 3. Materials and Methods

### 3.1. Materials

Indium tin oxide (ITO, sheet resistance: 7 ohm/square), cesium iodide (CsI, purity 99.999%) and tin (II) iodide (SnI_2_, purity 99.999%) were purchased from Advanced Election Technology Co., Ltd. (Yingkou, China). Formamidinium iodide (FAI, purity 99.5%), methylammonium iodide (MAI, purity 99.5%), bathocuproine (BCP) and C60 were purchased from Xi’an Yuri Solar Co., Ltd. (Xi’an, China). Tin fluoride (SnF_2_, purity 99.9%) was purchased from Shanghai Macklin Biochemical Technology Co., Ltd. (Shanghai, China). PE-DOT:PSS 4083 (1.3–1.7 wt%) was purchased from Heraeus Precious Metals GmbH (Hanau, Germany). Chlorobenzene (CBZ, anhydrous, purity 99.8%), dimethyl sulfoxide (DMSO, anhydrous, purity 99.9%) and *N*,*N*-dimethylformamide (DMF, anhydrous, purity 99.8%) were purchased from Beijing J&K scientific LLC (Beijing, China). Silver (Ag, purity 99.99%) was purchased from Beijing New Metal Materials Technology Co., Ltd. (Beijing, China). All purchased materials were used directly without any further purification.

### 3.2. Fabrication of Device

Perovskite preparation: The FA_0.75_MA_0.25_SnI_3_ perovskite precursor solution was prepared by mixing FAI (103.18 mg, 0.6 mmol), MAI (31.79 mg, 0.2 mmol), SnI_2_ (298 mg, 0.8 mmol) and SnF_2_ (12.54 mg, 0.08 mmol) in 1 mL anhydrous DMF and anhydrous DMSO mixed solution (DMF:DMSO = 4:1). The final tin element concentration was 0.8 M. Then, it was stirred overnight at room temperature and filtered with a 0.22 um polytetrafluoroethylene (PTFE) filter before use. All these preparation processes were carried out in nitrogen filled glovebox (O_2_ < 0.1 ppm, H_2_O < 0.1 ppm).

Device fabrication: The etched ITO glass was scrubbed several times with a tooth-brush dipped in Hellmanex cleaning solution. Then, it was sequentially ultrasonically cleaned in deionized water, ethanol and isopropyl alcohol for 15 min each. Before use, the ITO substrates were dried with nitrogen and cleaned with UV-ozone treatment for 20 min. For the preparation of the hole transport layer, the PEDOT:PSS was filtered using a 0.45 μm polyether sulfone (PES) filter first and then spin-coated on ITO surface at 4000 rpm for 30 s, and annealed at 150 °C for 30 min in ambient air. The substrates were transferred to a nitrogen glovebox for the following fabrication process. The ambience of the N_2_ filled glovebox was maintained at O_2_ < 0.1 ppm, H_2_O < 0.1 ppm. A 40 µL perovskite precursor solution was spin-coated on the PEDOT:PSS surface at 5000 rpm for 60 s; 120 µL chlorobenzene (CBZ) was quickly dripped onto the substrate at 13 s from the start, followed by 15 min annealing at 90 °C. Cesium iodide (CsI, 0.1 nm/s) of different thicknesses (0 nm, 7.5 nm, 10 nm and 12.5 nm), C_60_ (35 nm at 0.1 nm/s) and BCP (8 nm at 0.1 nm/s) were deposited by thermal evaporation under high vacuum of 10^−4^ Pa. Finally, 80 nm thick Ag electrode was thermal evaporated as cathode under high vacuum of 10^−4^ Pa. The active area of perovskite solar cells was 5 mm^2^.

### 3.3. Characterizations

*J*-*V* Characterization: The current–voltage characteristics were obtained using a Keithley 2400 (Keithley, Cleveland, OH, USA)Source Meter under simulated one-sun AM 1.5G illumination (100  mW/cm^2^) with a solar simulator (Newport, Irvine, CA, USA, AAA); the light intensity was calibrated using a KG-5 Si diode. The device current density–voltage curve was measured by forward scan ranging from −0.1 V to 0.5 V with an interval of 5 mV/s and reverse scan ranging from 0.5 V to −0.1 V with an interval of 5 mV/s. Light intensity-dependent *J*-*V* characteristics were measured using the same solar simulator setup, with varying sun intensity from 10 to 100 mW/cm^2^. The current–voltage curve under dark conditions (dark current curve) was tested by the same device that simulates dark conditions. The capacitance-voltage (*C*-*V*) curves were measured using High Energy Resolution Analysis Deep level Transient Spectroscopy FT1230 HERA DLTS (PhysTech, Kassel, Germany) and the sample structure, ITO/PEDOT:PSS/PVSK (CsI) with 1 mm diameter Ag (thickness is 80 nm), was deposited on PVSK. Spin-coated films on the ITO substrate were imaged using a scanning electron microscope (GeminiSEM 300, ZEISS, Oberkochen, Germany) under an accelerating voltage of 15 kV. Grain diameters in the SEM pictures were analyzed using a Nano measurer. Steady state photoluminescence (PL) was characterized using a Nanolog FL3-iHR320 (HORIBA, Irvine, CA, USA); the excitation wave-length was set at 520 nm during the test and the structure was Pure glass/PVSK (CsI)/C_60_, with 35 nm C_60_. X-ray diffraction patterns (XRD) were analyzed utilizing a Bruker X-ray diffractometer (D8 focus) using Cu Kα irradiation and scan ranging from 10 degrees to 70 degrees with 0.02 step at room temperature; the sample structure was also ITO/PEDOT:PSS/PVSK (CsI). The contact angles of perovskite films with different thickness of CsI were tested by contact angle measurement (SCI 2000A, Global Hengda Technology Co., LTD, Beijing, China).

Device Stability Testing: The PVSK/CsI (10 nm) devices were placed in an N_2_-filled glovebox (H_2_O < 0.1 ppm and O_2_ < 0.1 ppm) at room temperature and the *J–V* curves were measured at different time points. All the performance characterization was carried out in ambient atmosphere without any encapsulation of the devices.

## 4. Conclusions

In summary, we have demonstrated an effective strategy for boosting the fill factor and efficiency of tin-based perovskite cells using thin CsI layer modification. With the thin CsI modification layer, the perovskite film showed increased grain size. Meanwhile, the thin CsI layer chemical reacted with the unpaired/unreacted SnI_2_ to form the more stable and hydrophobic Cs/FA/MA-based perovskites. These resulted in a lower non-radiative recombination loss and decreased trap density of the tin perovskite films. Consequently, the series resistance of the devices was decreased; the shunt resistance of the devices was increased. Therefore, the fill factor and device efficiency were greatly increased. With 10 nm CsI film between the perovskite and C_60_ layers, the champion CsI-PSCs cell showed a fill factor of 63% and a PCE of 6.1%. Moreover, the CsI-PSCs cells showed significantly improved device stability. Our study reveals that, through moderate interface engineering, stable tin-based perovskite solar cells can be achieved.

## Figures and Tables

**Table 1:** Photovoltaic performance parameters of tin perovskite cells with varying CsI thickness.

CsI (nm)	FF (%)	*J*_sc_ (mA/cm^2^)	*V*_oc_ (V)	PCE (%)	*R*_s_ (Ω)	*R*_sh_ (Ω)
0	44.4	19.1	0.43	3.7	99.1	955.4
7.5	53.0	20.4	0.34	3.7	55.8	1009.7
10	64.0	21.8	0.34	4.7	33.4	1451.9
12.5	43.5	21.4	0.29	2.7	62.1	557.4

## Data Availability

The data that support the findings of this study are available from the corresponding authors upon reasonable request.

## Supplementary Materials

The following supporting information can be downloaded at: https://www.mdpi.com/article/10.3390/molecules28176414/s1, Figure S1. Schematic view of the inverted FA_0.75_MA_0.25_SnI_3_ perovskite solar cell structure with/without CsI thin passivation layer. Figure S2. (a) Light current density–voltage (light *J-V*) characteristic curves. (b) Dark current density–voltage characteristic curves (dark J–V) of the FA_0.75_MA_0.25_SnI_3_ Sn based perovskite solar cells with thin CsI passivation layers (0 nm, 7.5 nm, 10 nm, 12.5 nm). Figure S3. Capacitance–voltage (C-V) characteristic curves of the FA_0.75_MA_0.25_SnI_3_ Sn based perovskite films spin-coated on PEDOT:PSS layer with varied thickness CsI (a) 7.5 nm and (b) 12.5 nm. Figure S4. Top-view SEM images of the FA_0.75_MA_0.25_SnI_3_ perovskite films spin-coated on PEDOT:PSS layer with varied thickness CsI: (a) 7.5 nm and (b) 12.5 nm. Figure S5. Energy Dispersive Spectrometer (EDS) spectrum of the FA_0.75_MA_0.25_SnI_3_ perovskite films spin-coated on ITO substrate (a) without CsI layer modification (control); (b) with CsI layer modification (W/CsI). Figure S6. X-Ray Diffraction (XRD) patterns of FA_0.75_MA_0.25_SnI_3_ perovskite films spin-coated on PEDOT:PSS layer with varied thickness CsI (0 nm, 7.5 nm, 10 nm, 12.5 nm). Figure S7. Contact angle measurement of deionized water on FA0.75MA0.25SnI3 Sn based perovskite films spin-coated on PEDOT:PSS layer with varied thickness CsI (7.5 nm, 12.5 nm).
