# Supplementary material for: The Defect Passivation of Tin Halide Perovskites Using a Cesium Iodide Modification
Source: Molecules. 2023 Sep 3;28(17):6414. doi: 10.3390/molecules28176414 (PMC10490360; doi:10.3390/molecules28176414)
Supplement: Supplementary file 1 [file molecules-28-06414-s001.zip › molecules-2576895-supplementary.pdf]

Support Information

The Defect Passivation of Tin Halide Perovskites Using a Cesium Iodide Modification

Linfeng He [1], Jin Cheng [1,*], Longjiang Zhao [2], Xinyao Chen [1,3], Xiaoping Zou [1], Chunqian Zhang [1] and Junming Li [1,4]

1 Beijing Key Laboratory for Sensor, School of Applied Science, Beijing Information Science and Technology University, Beijing 100101, China.
2 College of Engineering, Qufu Normal University, Rizhao 276826, China.
3 School of Instrument Science and Opto-Electronics Engineering, Beijing Information Science and Technology University, Beijing 100101, China.
4 Fujian Key Laboratory of Electrochemical Energy Storage Materials, Fuzhou University,
Fuzhou 350002, China
* Correspondence: chengjin@bistu.edu.cn

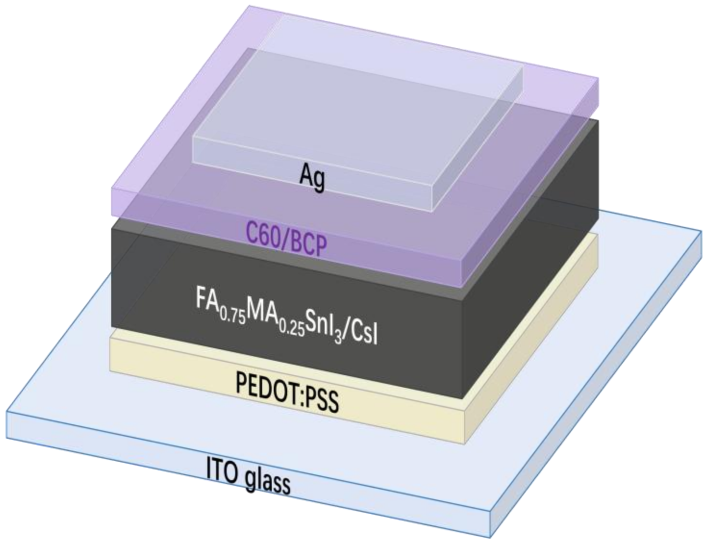

Figure S1. Schematic view of the inverted $FA_{0.75}MA_{0.25}SnI_3$ perovskite solar cells structure with/without CsI thin passivation layer.

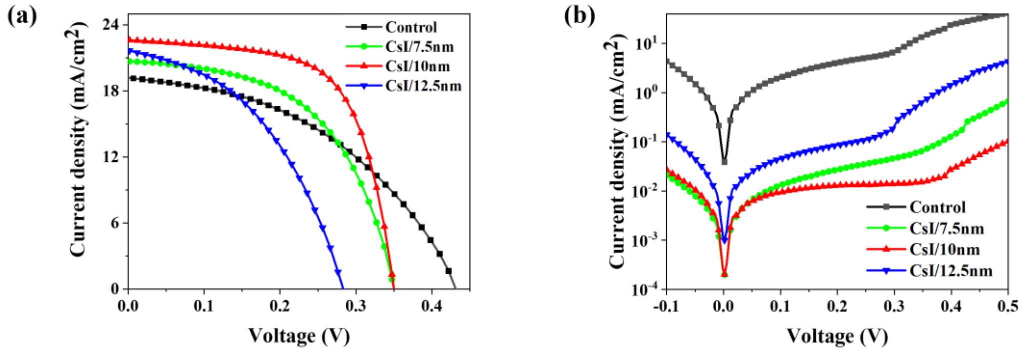

Figure S2. (a) Light current density-voltage (light *J-V*) characteristic curves; (b) Dark current density-voltage characteristic curves (dark J-V) of the $FA_{0.75}MA_{0.25}SnI_3$ Sn based perovskite solar cells with thin CsI passivation layers (0 nm, 7.5 nm, 10 nm, 12.5 nm).

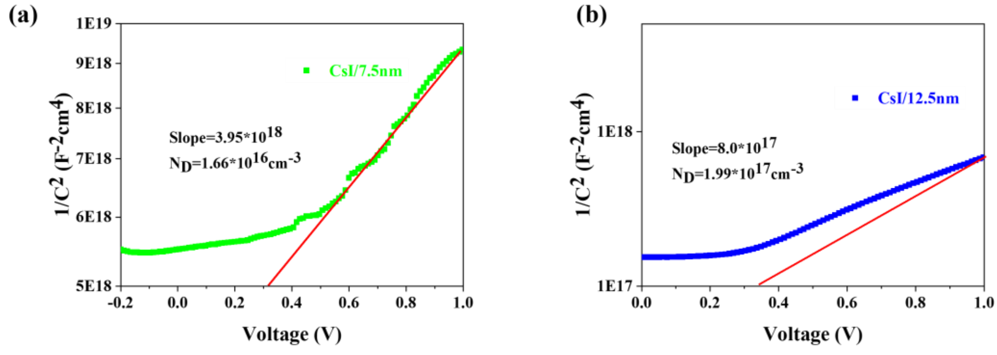

Figure S3. Capacitance-Voltage (*C-V*) characteristic curves of the $FA_{0.75}MA_{0.25}SnI_3$ Sn based perovskite films spin-coated on PEDOT:PSS layer with varied thickness CsI (a) 7.5 nm and (b) 12.5 nm.

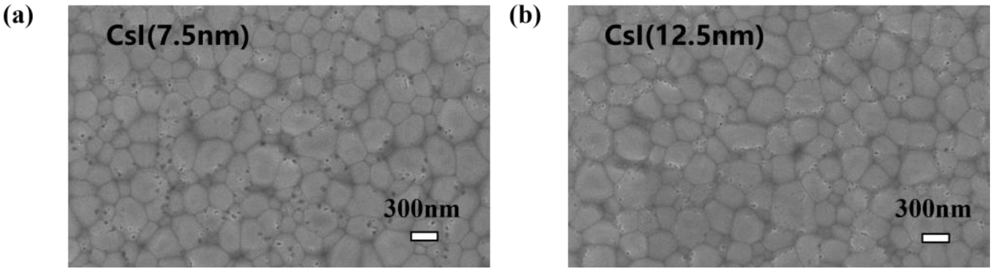

Figure S4. Top-view SEM images of the $FA_{0.75}MA_{0.25}SnI_3$ perovskite films spin-coated on PEDOT:PSS layer with varied thickness CsI: (a) 7.5 nm and (b)12.5 nm.

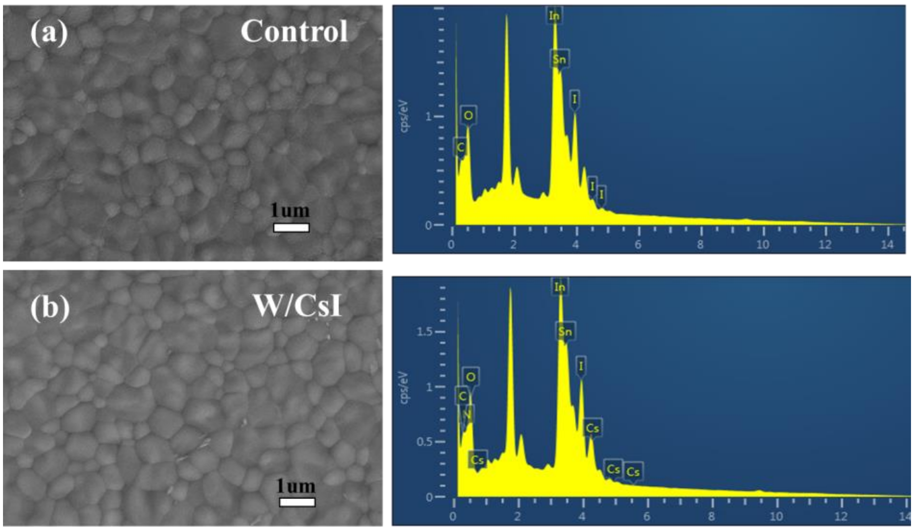

Figure S5. Energy Dispersive Spectrometer (EDS) spectrum of the $FA_{0.75}MA_{0.25}SnI_3$ perovskite films spin-coated on ITO substrate (a) without CsI layer modification (control); (b)with CsI layer modification (W/CsI).

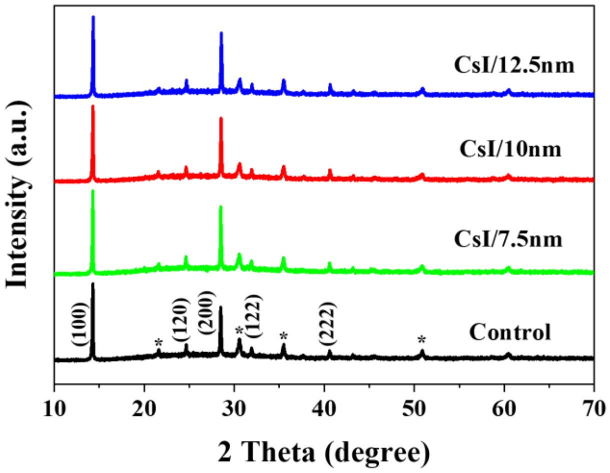

Figure S6. X-Ray Diffraction (XRD) patterns of $FA_{0.75}MA_{0.25}SnI_3$ perovskite films spin-coated on PEDOT:PSS layer with varied thickness CsI (0 nm, 7.5 nm, 10 nm, 12.5 nm) and * is represent the peak of ITO.

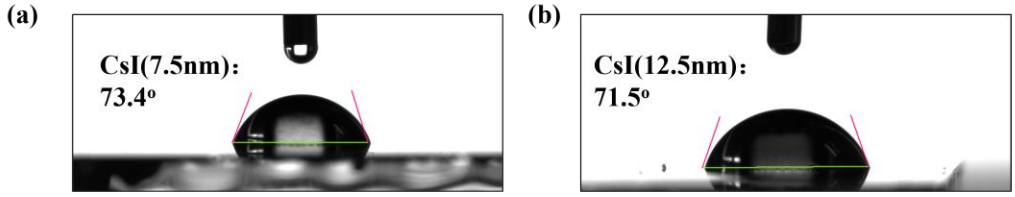

Figure S7. Contact angle merasument of deionized water on $FA_{0.75}MA_{0.25}SnI_3$ Sn based perovskite films spin-coated on PEDOT:PSS layer with varied thickness CsI (7.5 nm, 12.5 nm).
